# Improving Oratory Skills: An “American Idol” Presentation Competition for Residents

**DOI:** 10.7759/cureus.3049

**Published:** 2018-07-25

**Authors:** David A Hill, Jean-Carlos Jimenez, Mitchell R Price, Stephen M Cohn

**Affiliations:** 1 Plastic and Reconstructive Surgery, Houston Methodist Hospital, Houston, USA; 2 Surgery, Northwell Health at Staten Island University Hospital, Staten Island, USA; 3 Pediatric Surgery, Northwell Health at Staten Island University Hospital, Staten Island, USA; 4 Surgery, Staten Island University Hospital, Queens Village, USA

**Keywords:** public speaking, presentations, residency education, resident competition

## Abstract

Background

It is essential for physicians to master the ability to deliver high-quality oral presentations. Despite this, little time is dedicated throughout residency for training and refining this important skill. In order to solve this issue, we set out to design and implement a course which will improve the oratory skills of the resident physicians.

Methods

Senior surgical residents (postgraduate years three and four) were involved in a single-elimination tournament with the audience voting for the top presenters. Faculty provided feedback on oration, slide layout and overall presentation format throughout the course. Baseline and post-course survey responses were evaluated to assess a change in presentation skills after the “oratory course”.

Results

Seven senior residents participated as competitors. Seventeen other junior and chief residents (postgraduate years 1, 2 and 5) were involved as audience members along with several attending physicians, physician assistants and medical students. Both the presenters and audience appreciated a statistically significant improvement in communication skills and slide layout (p < 0.01).

Conclusion

The use of a structured course in public speaking and presentation skills proved to be effective in developing oratory skills in surgical residents when used in conjunction with an entertaining format.

## Introduction

In addition to the everyday responsibility of making life-altering decisions, doctors are also required to be educators and at times orators. It is through teaching and mentorship that physicians aid in shaping the future of medicine. Therefore, it becomes essential for doctors to master the ability to deliver high-quality oral presentations whether they are for large audiences or on rounds [1, 2]. We set out to improve our surgical trainee’s oratory and presentation skills via the design and implementation of a novel competitive course in public speaking. We hypothesized that implementing an interactive oratory course for trainees would result in an appreciable improvement in presentation skills.

## Materials and methods

We developed and implemented an academic competition in public speaking between our surgical trainees at Staten Island University Hospital. The contest was structured similar to an “American Idol” elimination program. Residents presented randomly assigned topics over a four week period. After each presentation, faculty provided constructive criticism related to time management, slide quality and content and the effectiveness of the lecture delivered. At the end of each round, the audience, consisting of residents, medical students, nurses, physician assistants and faculty, voted anonymously for the residents they believed should advance to the next week.

The first session consisted of all presenters giving a five-minute discussion on a random topic of their choosing. Each presenter was limited to five PowerPoint slides. At the end of each presentation, judges were allowed to voice several comments and critiques. This round was solely for practice and did not include voting or elimination. Upon completion of this initial round, both the presenters and the audience were surveyed in order to determine impressions concerning the baseline capabilities of the competitors.

**Figure 1:**
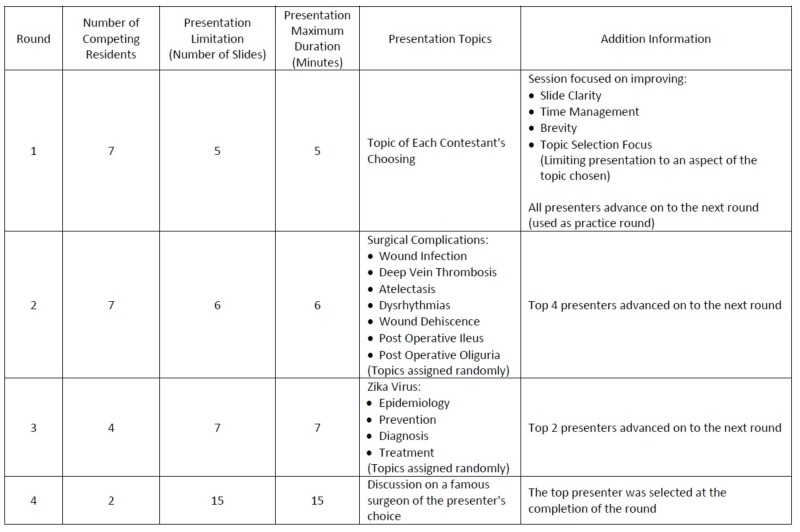
Course outline describing weekly competition guidelines and topics.

In the second week, each resident presented a preselected topic which was assigned by picking the subject out of a hat. The topics were determined by the course organizer and were related to a common theme – postoperative complications (Figure 1). The contestants were given six minutes to present a maximum of six slides. Unlike the practice round, judges were limited to one question and one comment each. The order of presentation was determined by random chance. The audience was asked to rank each resident presenter. The top four proceeded on to the next round.

The third round proceeded similar to that of the second. All topics were selected out of a hat as was the presentation order. Again, at the end of each resident’s speech the faculty panel shared their opinions. This third session allotted seven minutes and seven slides per contestant. The top two presenters advanced on to the fourth and final round.

In the last session, each presenter was given 15 minutes to present a maximum of 15 slides on a topic of their choosing within a designated subject matter. The order of presentation this time was determined by lottery, with the winner deciding the order. Upon completion of the course, and prior to disclosing the overall winner, both the audience and all of the resident competitors filled out a final survey which was identical to the initial survey.

Demographic and other characteristics were summarized by the number and percentages of survey responses. Differences in communication level, slide clarity and feedback usefulness between pre- and post-competition participants and audience members were compared using the Kruskal-Wallis test. All statistical tests were two-sided and conducted at the 0.05 level of significance. Data analyses were conducted using the SAS® System Version 9.3 (SAS Institute Inc., Cary, NC). Study data was collected and managed using research electronic data capture (REDCap) tools hosted at Staten Island University Hospital [3].

Prior to the initiation of this competition, an institutional review board waiver was submitted and exemption granted on the ground that this was considered a quality improvement initiative and fell under the category of research being conducted in established or commonly accepted educational settings, involving normal educational practices.

## Results

**Figure 2:**
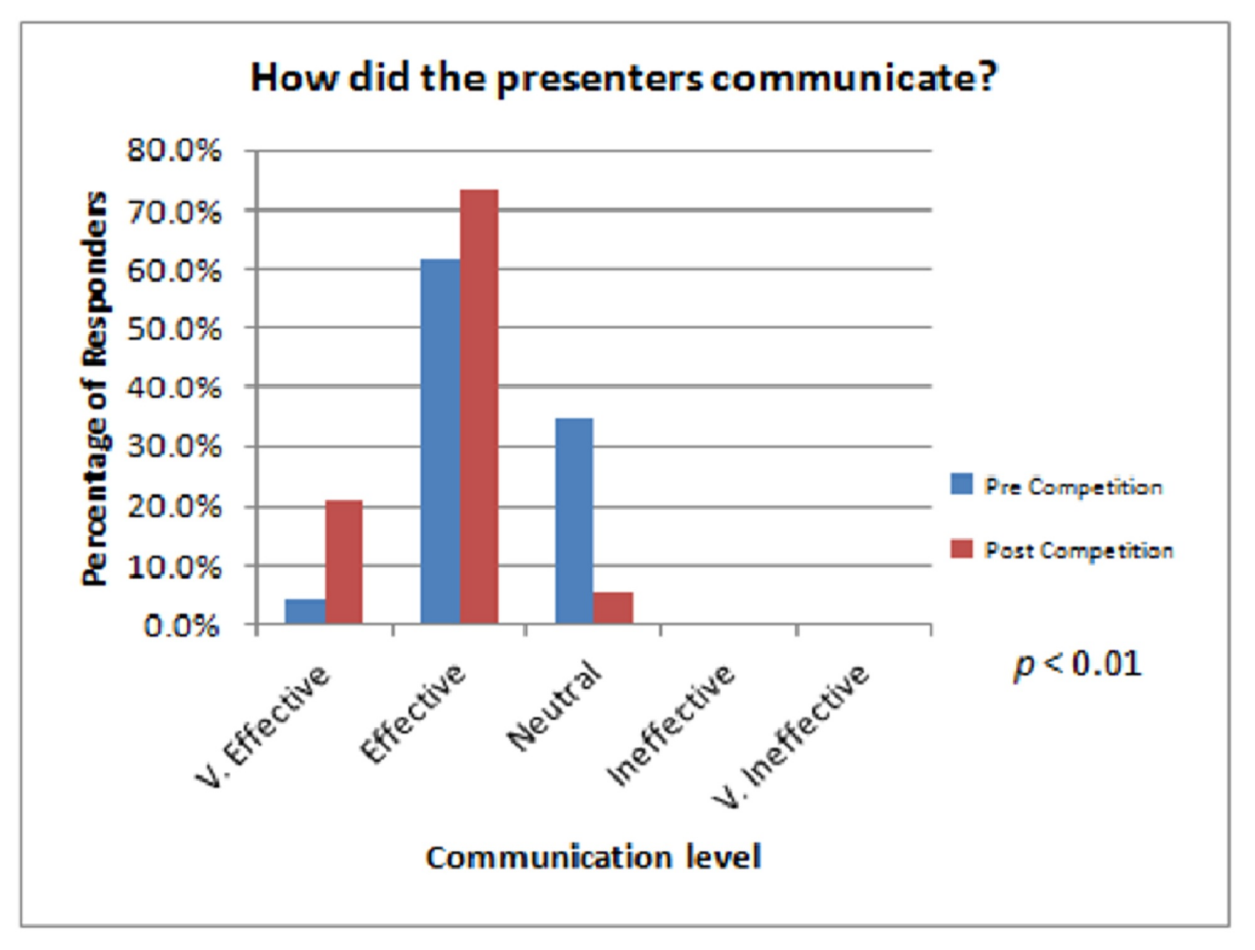
Audience survey results concerning how well the presenters communicated both before and after the course. V = Very

All seven senior surgical residents competed in the competition (100% senior resident participation rate). Three of these were third-year residents and four were fourth years. A total of 17 junior and chief residents, postgraduate year 1, 2 and 5 respectively, were not involved as competitors. Therefore, 29% of the residency program was selected to compete. The audience consisted of various amounts of other non-competing surgical residents, attending physicians, physician assistants, and medical students. Twenty-six audience members filled out the baseline survey during the first week’s session. Nineteen completed the final audience survey during the last round. Attending judges were not included in the audience polling (Figure 2).

Throughout the presentation course, there was a noticeable improvement in the quality of the residents’ presentations. The presenters felt their communication skills were much more effective at the end of the course. Also noted was the improvement in understanding oratory time management and slide preparation. An unexpected course outcome was toward the final rounds as the majority of the residents began providing constructive criticism to their colleagues.

**Figure 3:**
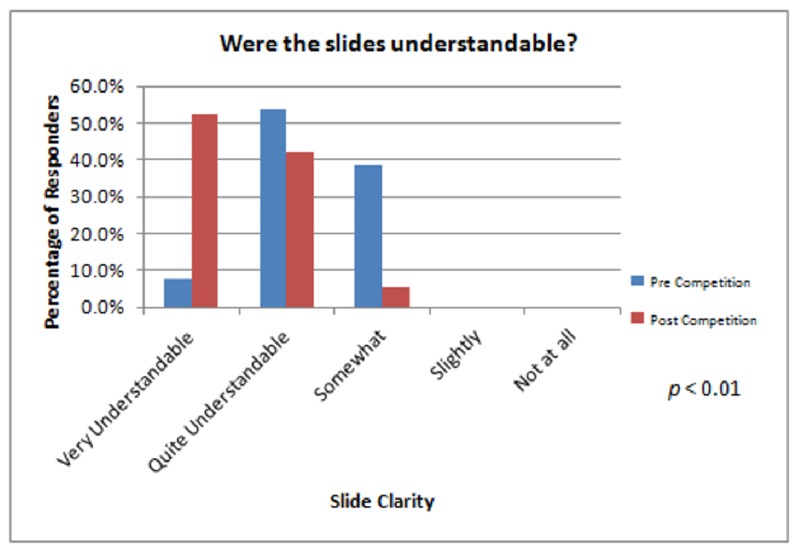
Audience survey results concerning how understandable the presenters' slides were both before and after the course.

We found a statistically significant difference (p < 0.01) in two areas pertaining to the course experience. First, the audience noted that the presenters communicated markedly better by the end of the course. Second, the slide layout and clarity were significantly improved by week four (Figure 3).

## Discussion

Residents need to master the art of presenting as it is a crucial part of their future career [1, 4]. The ability to deliver speeches with clarity and brevity is a cornerstone of medical education [2]. Few residents come into a surgical residency program with these skills already honed. Reliance on repetition over time to attain these skills, without actual instruction, may not work for all residents, and can effectively act as a deterrent in their surgical careers [5].

**Figure 4:**
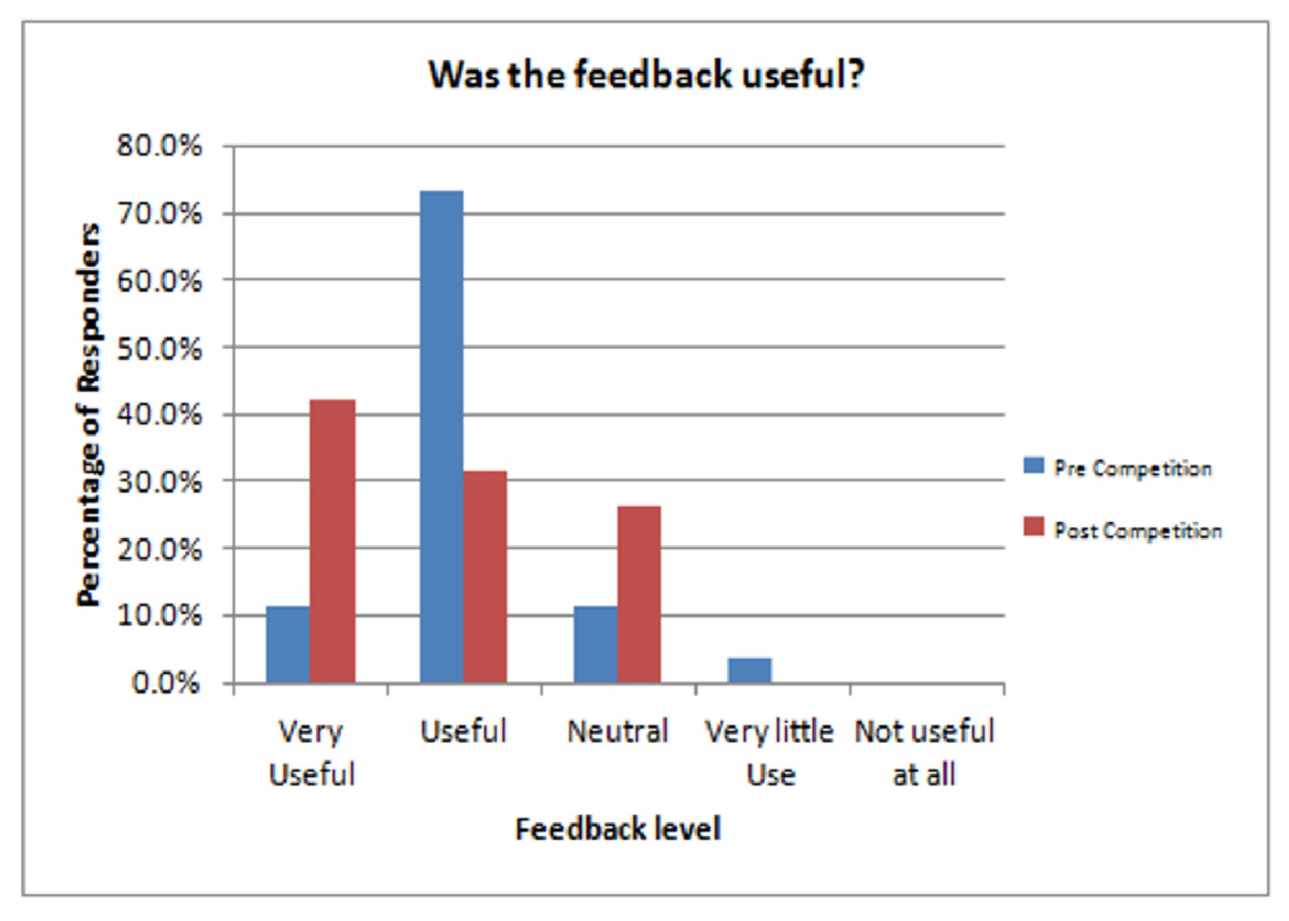
Audience survey results concerning how useful the feedback, given at the end of each presentation, was.

The creation and implementation of a presentation course for postgraduate trainees resulted in an entertaining experience that significantly improved both presentation slide layout and clarity and presenter communication. In this competition model, using a small group of participants in front of a panel of faculty and colleagues, trainees demonstrated improvement in the areas of preparation and fluidity. By the end of the course, over 70% of both the audience and resident participants reported the feedback to be very constructive (Figure 4). Participants also acknowledged that their level of expertise in giving a presentation improved throughout the course. Contestants elevated the quality of their slides, the content of their lectures and their overall presentation delivery.

Perhaps one of the most beneficial aspects of the course was presentation skills gained from observational learning. Points emphasized through criticism from faculty were subsequently absorbed by the audience to be utilized in future presentations. One such example was time management. Throughout the very first two presentations, time utilization was poor and the presenters used only three out of the five minutes allotted. The remaining presenters recognized this fault and ensured they would not receive similar criticism. This alone resulted in the presenters slowing down the tempo of their lecture, speaking more clearly and better emphasizing important aspects of their presentations. Because of this observational learning, it is hopeful that in years to come, the experience of the junior residents as observers will lead to a heightened quality of presentation when they are senior residents.

The biggest limitation to the study would be the sample size involved, which consisted of only 19–26 survey responders. This small sample size makes it difficult to demonstrate statistical significance. However, the smaller amount of participants helped to contribute to making the course feel more personable. Instead of audience members being lost in a sea of people they were in fact a crucial portion of the success of the course in that they were essential in providing feedback to the presenters. This also potentially led to more audience members paying attention and learning more throughout the competition by not being distracted by substantial amounts of people around them.

After comparing pre-competition lectures to ones given in recent weeks, this course notably builds confidence and elevates oration skills through objective analysis, constructive criticism and friendly competition.

## Conclusions

The development and implementation of a structured course in public speaking and presentations proved to be effective in developing oratory skills in surgical residents.
